# Bridging the gap: improving correspondence between low-field and high-field magnetic resonance images in young people

**DOI:** 10.3389/fneur.2024.1339223

**Published:** 2024-02-23

**Authors:** Rebecca Cooper, Rebecca A. Hayes, Mary Corcoran, Kevin N. Sheth, Thomas Campbell Arnold, Joel M. Stein, David C. Glahn, Maria Jalbrzikowski

**Affiliations:** ^1^Department of Psychiatry and Behavioral Sciences, Boston Children’s Hospital, Boston, MA, United States; ^2^Department of Psychiatry, Harvard Medical School, Boston, MA, United States; ^3^Center for Brain and Mind Health, Yale School of Medicine, New Haven, CT, United States; ^4^Center for Neuroengineering and Therapeutics, University of Pennsylvania, Philadelphia, PA, United States; ^5^Department of Radiology, Perelman School of Medicine, University of Pennsylvania, Philadelphia, PA, United States; ^6^Olin Neuropsychiatry Research Center, Institute of Living, Hartford, CT, United States

**Keywords:** magnetic resonance imaging, low field MRI, pediatric neuroimaging, SynthSR, super-resolution

## Abstract

**Background:**

Portable low-field-strength magnetic resonance imaging (MRI) systems represent a promising alternative to traditional high-field-strength systems with the potential to make MR technology available at scale in low-resource settings. However, lower image quality and resolution may limit the research and clinical potential of these devices. We tested two super-resolution methods to enhance image quality in a low-field MR system and compared their correspondence with images acquired from a high-field system in a sample of young people.

**Methods:**

T1- and T2-weighted structural MR images were obtained from a low-field (64mT) Hyperfine and high-field (3T) Siemens system in *N* = 70 individuals (mean age = 20.39 years, range 9–26 years). We tested two super-resolution approaches to improve image correspondence between images acquired at high- and low-field: (1) processing via a convolutional neural network (‘SynthSR’), and (2) multi-orientation image averaging. We extracted brain region volumes, cortical thickness, and cortical surface area estimates. We used Pearson correlations to test the correspondence between these measures, and Steiger Z tests to compare the difference in correspondence between standard imaging and super-resolution approaches.

**Results:**

Single pairs of T1- and T2-weighted images acquired at low field showed high correspondence to high-field-strength images for estimates of total intracranial volume, surface area cortical volume, subcortical volume, and total brain volume (*r* range = 0.60–0.88). Correspondence was lower for cerebral white matter volume (*r* = 0.32, *p* = 0.007, *q* = 0.009) and non-significant for mean cortical thickness (*r* = −0.05, *p* = 0.664, *q* = 0.664). Processing images with SynthSR yielded significant improvements in correspondence for total brain volume, white matter volume, total surface area, subcortical volume, cortical volume, and total intracranial volume (*r* range = 0.85–0.97), with the exception of global mean cortical thickness (*r* = 0.14). An alternative multi-orientation image averaging approach improved correspondence for cerebral white matter and total brain volume. Processing with SynthSR also significantly improved correspondence across widespread regions for estimates of cortical volume, surface area and subcortical volume, as well as within isolated prefrontal and temporal regions for estimates of cortical thickness.

**Conclusion:**

Applying super-resolution approaches to low-field imaging improves regional brain volume and surface area accuracy in young people. Finer-scale brain measurements, such as cortical thickness, remain challenging with the limited resolution of low-field systems.

## Introduction

Magnetic resonance imaging (MRI) has facilitated our current understanding of the processes underlying mental health and illness, and is routinely used in pediatric clinical and neuroimaging research (1–3). Ongoing advancements in neuroimaging technology and the development of state-of-the-art statistical techniques have helped to improve our understanding of the etiology of youth mental health disorders, and novel brain-based risk markers show promise in identifying and providing prognostic information for at-risk individuals (4–6). However, there are several challenges associated with conventional high-field MRI systems that limit the translation of these promising findings, prohibiting their widespread use and incorporation in community settings.

Low-field-strength MRI (LF-MRI) is a promising alternative that addresses several limitations inherent in high-field-strength systems. LF-MRI machines typically operate with magnetic fields below 0.3 Tesla (compared to high-field systems at 1.5–3T), and carry the advantages of substantially lower installation and maintenance costs, reduced power consumption, smaller space requirements, and do not require cryogenic cooling (7, 8). Several portable systems have been developed, and can be installed in settings with limited or unreliable power supply with minimal operator expertise. The successful deployment of LF-MRI within intensive care units (9–13), vehicles (14), consulting offices (15) and in low-resource settings (16, 17) demonstrates the increased accessibility offered by low-field technology. This improved accessibility carries the potential to reduce long-standing disparities in access to diagnostic imaging within the United States (18, 19). In addition, the lower-intensity acoustic noise and open scanner designs in LF systems provide advantages for pediatric populations, improving scanning success rates and reducing the need for child sedation (20, 21). In a sample of 42 healthy children aged 6 weeks to 16 years of age, superior completion rates were achieved in a low-field (64mT) LF system compared to conventional high-field (3T) MRI system [89% compared to 75%; (22)]. In addition, global estimates of cortical volume showed strong correspondence between low-field- and high-field acquired images, with low-field images successfully recapitulating global gray-matter age-associations (22). However, components of cortical volume (e.g., cortical thickness, surface area), as well as cerebral white matter and subcortical volume, show diverse trajectories of growth across development (23–26), are believed to reflect distinct biological underpinnings (27–29), and have differing genetic influences (27, 30). For LF-MRI to be feasibly used at scale in young people, we need to test the ability of this technology to accurately estimate diverse components of global and regional brain structure.

One of the major drawbacks of LF systems is a low signal-to-noise ratio (SNR), resulting in poorer image resolution and quality (7, 8). However, recent developments in ‘super-resolution’ approaches, defined as methods that reconstruct high-resolution images from a series of low-resolution images, may help to address these shortcomings. Multi-orientation image averaging, which involves reconstruction of several low-resolution scans taken in orthogonal slice directions (i.e., axial, sagittal, and coronal), has been found to significantly improve signal-to-noise ratio within neonatal samples (31, 32). Alternative super-resolution approaches that use state-of-the-art machine-learning techniques, such as convolutional neural networks (CNNs), also improve image resolution within LF systems (33). Initial work in a sample of adults (*N* = 11, *M* = 49.5 ± 14.1 years, seven males) demonstrated promising results with this approach, resulting in high correspondence between low-field (64mT) and high-field (1.5 or 3T) acquisitions across the cerebrum (33). While these studies show promise, we must ensure that such pipelines are developmentally appropriate for the acquisition and processing of low-field images among young people. It is unclear whether current super-resolution approaches, such as multi-orientation image averaging and machine-learning-based methods, are appropriate and effective for young populations, whether the effectiveness of these pipelines are moderated by additional factors (e.g., age, motion) or whether additional processing steps are necessary.

To test our ability to use data from low-field MRI scans in young people, we collected structural MRI data from low-field 64 mT and 3T MRI scanners from a community sample of young people (final *N* = 70, 9–26 years). We processed all scans through a standard structural neuroimaging pipeline (i.e., Freesurfer), and extracted measures of cortical and subcortical volume, cortical thickness, and surface area. We then used these extracted measures to conduct and compare correlational analyses of two super-resolution processing strategies. First, we examined correspondence between brain measures extracted from low-field scans to brain measures extracted from 3T scans. Second, we tested whether synthesizing super-resolution MP-RAGE images from the low-field scans via a CNN approach improved the correspondence between low-field and high-field images. Third, because we collected several pairs of low-field T1- and T2-weighted scans, we examined how multi-orientation image averaging improved correspondence with measures derived from high-field scans. We tested how implementing a combination of both multi-orientation and CNN-processing approaches influenced these relationships. Finally, we examined how other factors, i.e., age and motion (34–36), are related to our ability to capture high-field quality measurements with low-field scans.

## Materials and methods

### Participants

In the current study, we recruited a community sample of 77 young people (9–26 years, *N* < 18 years = 18) from the Boston metro area. Inclusion/exclusion of data to obtain the final sample [*N* = 70, mean (SD) age = 20.39 (4.7) years] is detailed in Supplementary Figure 1. Demographic information for the final sample is reported in Table 1. Exclusion criteria were a history of a brain infection, presence of a neurodegenerative disorder (e.g., Parkinson’s disorder), presence of a neurodevelopmental disorder that might interfere with completion of study procedures, endorsement of a major mental disorder other than attention deficit-hyperactivity disorder (ADHD) or a past episode of depression, or any MRI contraindications. Given the increased prevalence of ADHD in youth (37, 38) and our desire to produce generalizable results, we did not exclude those with a diagnosis of ADHD in this sample. Participants completed the DSM Cross-Cutting Symptom Measure for Youth (39) and the Beck Depression Inventory (40) to assess sub-clinical symptoms of psychopathology. All participants provided written consent (if ≥18 years of age) or assent with written parental consent. Procedures were approved by the Institutional Review Board of Boston Children’s Hospital.

**Table 1:** Demographic features of the sample.

		*F*	*M*	Total
Total N		37	33	70
Mean Age (SD)		19.7 (4.9)	21.17 (4.5)	20.39 (4.7)
Age Range		9–26.7	9.8–26.9	9–26.9
Mean CCSM Total Score (SD)		9.11 (7.3)	8.82 (7.6)	8.97 (7.4)
Mean BDI Total Score (SD)		5.31 (7.2)	5 (5.8)	5.2 (6.7)
Race N (%)	American Indian/Alaskan Native	1	0	1 (1.4%)
	Asian	11	11	22 (31.4%)
	Black	3	1	4 (5.7%)
	White	22	20	42 (60%)
	Native Hawaiian/Pacific Islander	0	0	0 (0%)
	Interracial	0	1	1 (1.4%)
Ethnicity N (%)	Hispanic/Latino	3	4	7 (10%)
	Not Hispanic/Latino	34	29	63 (90%)

BDI, Beck Depression Inventory; CCSM, DSM self-rated Cross-Cutting Symptom Measure for Youth.

### MRI acquisition

**Figure 1:**
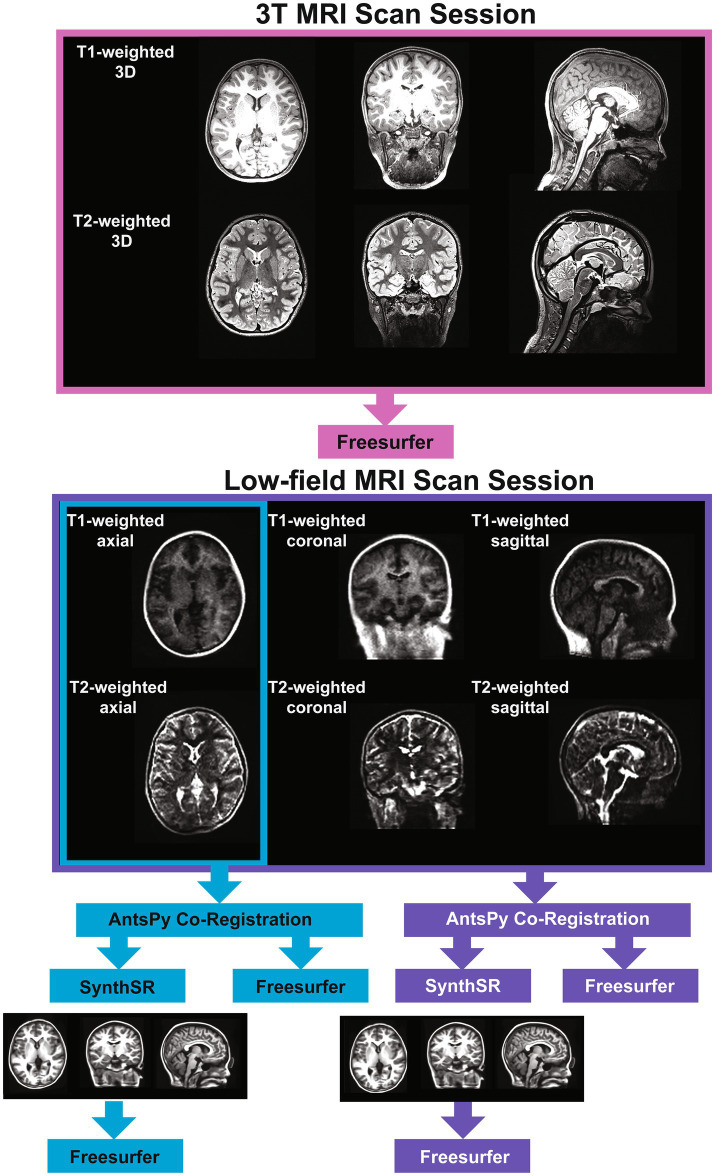
Image acquisition and processing pipeline.

We collected T1- and T2-weighted brain MRI scans for each participant using a Siemens Magnetom Prisma 3-Tesla scanner in a dedicated research MRI suite at Boston Children’s Hospital Brookline Place (total scan duration 12.61 min, scan resolution 0.8 × 0.8 × 0.8 mm). We also used a Hyperfine Swoop 64 mT scanner (total scan duration 52.25 min, scan resolution 1.6 × 1.6 × 5 mm) to collect two pairs of low-field T1- and T2-weighted scans using 3-dimensional techniques in sagittal, axial, and coronal orientations (see Figure 1). Scan parameters were determined following vendor- and software-provided and optimized sequence recommendations and are detailed in Supplementary Table 1. An experienced radiologist reviewed all 3T scans and reported no incidental findings in this sample. We used MRIQC (41) to verify scan quality, which provides a rating (1 [unusable] – 4 [excellent]) for each scan. We excluded all 3T MRI scans with a rating of 1 or 2 (*N* = 1).

### MRI processing

We tested two super-resolution approaches to process low-field data. For the single-acquisition approach, we used the ANTsPy Python module (v 0.3.9) (42, 43) to resample and coregister the axial-orientation T1- and T2-weighted low-field scans for each participant. For the multi-orientation approach, all six T1- and T2-weighted low-field MRI pairs were resampled to a 1.5 mm^3^ voxel size, then co-registered and averaged to reconstruct a single, composite higher-resolution image with ANTsPy. We then processed the low-field images via the Hyperfine-specific version of SynthSR (v. 1.0) (44). SynthSR is a convolutional neural network that predicts a 1 mm isotropic three-dimensional MP-RAGE image given one or more low-resolution inputs. We then processed all low-field and 3T MR images using FreeSurfer (v. 7.3.2) (45). FreeSurfer is an open-source automated segmentation software package for neuroimaging. In short, the following steps are part of the FreeSurfer processing stream: transformation of images to standard Talairach space, intensity normalization, removal of non-brain tissue, segmentation of white matter and subcortical structures, and final segmentation of cortical surfaces. Once the data was processed, we used the Desikan atlas (46) to extract regional measures of cortical surface area, cortical thickness, and cortical volume. The Desikan atlas includes 68 regions for each measure, resulting in a total of 204 cortical measures. We obtained subcortical volume measures using FreeSurfer’s subcortical atlas (*N* = 38 regions). We also extracted global measures of subcortical gray matter volume, total surface area, mean cortical thickness, cerebral white matter volume, total brain volume, cortical volume, and estimated total intracranial volume from each scan. This resulted in five sets of MR scans for each participant: (1) a single-acquisition standard low-field scan; (2) a single-acquisition, low-field scan processed through SynthSR; (3) a multi-orientation standard low-field scan; (4) a multi-orientation, SynthSR-processed low-field scan; and (5) a standard 3T scan.

### Statistical analyses

To test the association between the participants’ low-field and high-field scans we calculated Pearson correlations for each global and regional neuroimaging measure. We then used Steiger *Z*-tests to compare the difference in correspondence between the standard single-acquisition approach and each super-resolution method. First, we tested whether image processing via SynthSR statistically improved the fidelity of the low-field scans to their corresponding high-field images. Second, we compared correlation strengths between standard single-acquisition (one T1/T2 pair) and multi-orientation (six T1/T2 pairs) scan series. Third, we compared the standard single-acquisition approach to multi-orientation (six T1/T2 pairs) images processed with SynthSR. Fourth, we compared single-acquisition scans processed with SynthSR to multi-orientation scans processed with SynthSR. We used two additional methods to further evaluate the correspondence between low-field and 3T scans. First, we calculated the correspondence between low-field and 3T scans using two-way mixed intraclass correlation coefficients. Consistent with previous work (47, 48), we defined correspondence for both Pearson correlations and ICC values as follows: poor, *r* < 0.4; fair, 0.4 ≤ *r* < 0.6; moderate, 0.6 ≤ *r* < 0.75; high, *r* ≥ 0.75. Additionally, we used Bland–Altman plots to assess the agreement between low-field scans with the 3T estimates for each brain measure. This involved plotting the difference between the 3T and low-field estimates against the mean of the two estimates. For adequate agreement, Bland and Altman recommend that 95% of the data points should lie within ±1.96 standard deviations of the mean difference (49). Within each set of analyses, we used false discovery rate to correct for multiple comparisons separately in our global and regional analyses (50).

In secondary analyses, we examined whether potential confounding factors (participant age and motion artifacts) were associated with individual-level agreement between low- and high-field brain measures. We first calculated the absolute difference between low- and high-field estimates of each brain measure for each individual. We then converted this difference to a Z-score for each individual, with a positive Z-score indicating above average difference between high and low-field estimates (i.e., larger discrepancy between low-field and 3T measures) and a negative *Z*-score indicating below average difference between high- and low-field estimates (i.e., smaller discrepancy between low-field and 3T measures). We then examined the relationship between this Z-score and participant age or motion, measured as framewise displacement. We used Steiger *Z*-tests to examine whether these relationships significantly differed across the super-resolution approaches examined in the main analysis. Within each set of analyses, we used false discovery rate to correct for multiple comparisons separately in our global and regional analyses (50).

## Results

### Correspondence between standard (single-orientation) low-field- and high-field-acquired images

**Figure 2:**
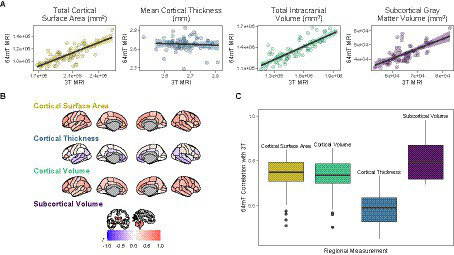
Correspondence between standard low-field (64mT) and high-field (3T) MR images. **(A)** Scatterplots of relationships between low-field and high-field images for global brain measures. Data points represent global estimates for each individual. Shading denotes 95% confidence intervals. **(B)** Pearson correlations for relationships between low-field and high-field images at the regional level. Shading represents mean correlation within each region across all individuals. **(C)** Distribution of correlation strengths for relationships between standard low-field and high-field images across each brain measure. Data points represent mean correlation across each region of interest.

**Figure 3:**
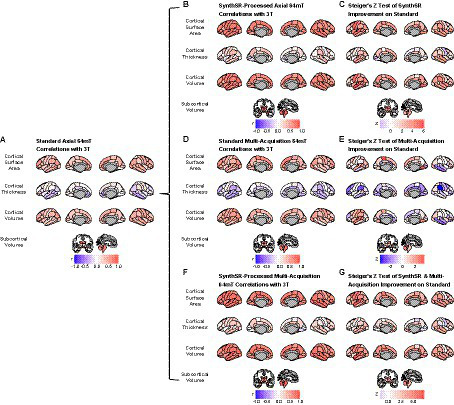
Comparison of super-resolution approaches in improving correspondence between low-field and high-field MR images. **(A)** Correlation of standard single-acquisition low-field images with high-field images for surface area, cortical volume, cortical thickness and subcortical volume. **(B)** Correlation of SynthSR-processed, single-acquisition low-field images with high-field images. **(C)** Steiger *Z*-test values for change in correspondence between standard and SynthSR-processed data. **(D)** Correlation of multi-orientation standard low-field images with high-field images. **(E)** Steiger *Z*-test values for change in correspondence between single-acquisition and multi-orientation approaches. **(F)** Correlation of SynthSR-processed, multi-orientation low-field images with high-field images. **(G)** Steiger *Z*-test values for change in correspondence between single-acquisition standard low-field images and multi-orientation, SynthSR-processed low-field images.

**Figure 4:**
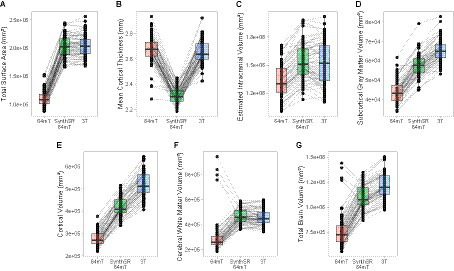
Comparison of individual-level global measurements across standard 64mT axial scans, SynthSR-processed 64mT axial scans, and traditional 3T scans. Boxplots show individual estimates for global brain measures **(A–G)** derived from standard axial 64mT scans (64mT), SynthSR-processed axial 64mT scans (SynthSR 64mT) and 3T scans (3T). Gray lines connect the same individuals across the three approaches.

For global brain measures, single (axial) acquisitions of T1/T2 low-field pairs showed high correspondence with high-field images for measures of total intracranial volume (Pearson’s *r* = 0.82, *p* = 5.58e-18, *q* = 1.82e-17) and total surface area (*r* = 0.76, *p* = 4.01e-14, *q* = 1.04e-13), and moderate correspondence for cortical volume (*r* = 0.67, *p* = 3.24e-10, *q* = 7.21e-10), subcortical gray matter volume (*r* = 0.66, *p* = 3.83e-10, *q* = 8.16e-10), and total brain volume (*r* = 0.60, *p* = 4.57e-08, *q* = 9.33e-08; see Table 2 and Figures 2, 3A). Correspondence was lower for cerebral white matter volume (*r* = 0.32, *p* = 0.007, *q* = 0.009) and non-significant for mean cortical thickness (*r* = −0.05, *p* = 0.664, *q* = 0.664). Similarly, intra-class correlation coefficients (ICC) for each brain measure showed high correspondence for total intracranial volume (ICC = 0.79, *p* = 1.11e-16, *q* = 3.04e-16), moderate correspondence for total surface area (ICC = 0.71, *p* = 1.65e-12, *q* = 4.15e-12) and cortical volume (ICC = 0.61, *p* = 1.08e-08, *q* = 2.34e-08), fair correspondence for subcortical gray matter volume (ICC = 0.66, *p* = 1.46e-10, *q* = 3.40e-10) and total brain volume (ICC = 0.56, *p* = 1.43e-07, *q* = 2.90e-07), poor correspondence for cerebral white matter volume (ICC = 0.24, *p* = 0.024, *q* = 0.036) and non-significant for mean cortical thickness (ICC = −0.05, *p* = 0.668, *q* = 0.701; see Table 3). When we examined the individual data distributions (Figure 4), we found that standard single-acquisition low-field images typically underestimated high-field values for each brain measure. Mean differences (low-field subtracting 3T) for surface area were − 45.8% [standard deviation (SD) 4.4], cortical thickness + 0.85% (5.5), cortical volume − 46.4% (5.3), subcortical volume − 32.5% (7.0), cerebral white matter volume − 33.8% (29.4), total brain volume − 37.0% (11.4) and intracranial volume − 10.1% (6.83). Bland–Altman analyses for agreement between low- and high-field scans (see Supplementary Figure 2) showed positive bias for total surface area (bias 9.35e04mm^2^, 95% limits of agreement 6.78e04-11.93e04), total brain volume (4.44e05 mm^3^ [1.62e5−7.25e5]), cortical volume (2.37e5 mm^3^ [1.59e5-3.22e5]), intracranial volume (1.57e05 mm^3^ [−0.55e5−3.70e5]), subcortical volume (2.12e04 mm^3^ [1.16e4-3.08e4]) and cerebral white matter volume (1.53e05 mm^3^ [−1.11e5–4.18e5]), indicating the low-field acquisition underestimated high-field data. Bias for cortical thickness was not significantly different from zero (−0.02 mm [−0.31–0.28]), indicating that, on average, low-field estimates neither over- or under-estimated the high-field data.

**Table 2:** Pearson correlation coefficients for the correspondence between low-field (64mT) and high-field (3T) MR images and comparisons across super-resolution approaches.

Brain measure	Standard Axial 64mT Correlations with 3T			SynthSR-Processed Axial 64mT Correlations with 3T			Steiger’s		
	*r*	*p*	*q*	*r*	*p*	*q*	*Z*	*p*	*q*
Total Surface Area	**0.76**	**4.01e-14**	**1.01e-13**	**0.90**	**9.01e-26**	**5.16e-25**	**3.73**	**1.94e-04**	**3.22e-04**
Mean Cortical Thickness	−0.05	0.664	0.697	0.14	0.241	0.286	1.43	0.153	0.186
Estimated Intracranial Volume	**0.82**	**5.58e-18**	**1.67e-17**	**0.85**	**1.74e-20**	**6.10e-20**	1.45	0.148	0.183
Subcortical Gray Matter Volume	**0.66**	**3.83e-10**	**8.32e-10**	**0.88**	**4.89e-24**	**2.57e-23**	**4.57**	**4.84e-06**	**8.97e-06**
Cortical Volume	**0.67**	**3.24e-10**	**7.28e-10**	**0.86**	**1.94e-21**	**7.17e-21**	**4.18**	**2.90e-05**	**5.08e-05**
Cerebral White Matter Volume	**0.32**	**0.007**	**0.010**	**0.92**	**1.23e-28**	**7.74e-28**	**8.75**	**2.18e-18**	**6.88e-18**
Total Brain Volume	**0.60**	**4.57e-08**	**9.60e-08**	**0.97**	**1.15e-43**	**1.81e-42**	**9.80**	**1.15e-22**	**5.59e-22**
Brain measure	Standard Axial 64mT Correlations with 3T			Standard Multi-Orientation 64mT Correlations with 3T			Steiger’s		
	*r*	*p*	*q*	*r*	*p*	*q*	*Z*	*p*	*q*
Total Surface Area	**0.76**	**4.01e-14**	**1.01e-13**	**0.58**	**1.07e-07**	**2.18e-07**	**−2.52**	**0.012**	**0.017**
Mean Cortical Thickness	−0.05	0.664	0.697	−0.25	0.038	0.051	−1.54	0.123	0.155
Estimated Intracranial Volume	**0.82**	**5.58e-18**	**1.67e-17**	**0.78**	**1.29e-15**	**3.39e-15**	**−2.36**	**0.018**	**0.026**
Subcortical Gray Matter Volume	**0.66**	**3.83e-10**	**8.32e-10**	**0.71**	**5.86e-12**	**1.42e-11**	0.94	0.349	0.393
Cortical Volume	**0.67**	**3.24e-10**	**7.28e-10**	**0.54**	**1.40e-06**	**2.68e-06**	−1.76	0.079	0.101
Cerebral White Matter Volume	**0.32**	**0.007**	**0.010**	**0.41**	**3.63e-04**	**5.73e-04**	**3.57**	**3.64e-04**	**5.73e-04**
Total Brain Volume	**0.60**	**4.57e-08**	**9.60e-08**	**0.69**	**3.30e-11**	**7.71e-11**	**3.49**	**4.84e-04**	**7.44e-04**
Brain measure	Standard Axial 64mT Correlations with 3T			SynthSR-Processed Multi-Orientation 64mT Correlations with 3T			Steiger’s		
	*r*	*p*	*q*	*r*	*p*	*q*	*Z*	*p*	*q*
Total Surface Area	**0.76**	**4.01e-14**	**1.01e-13**	**0.87**	**1.42e-22**	**6.38e-22**	**2.85**	**0.004**	**0.007**
Mean Cortical Thickness	−0.05	0.664	0.697	0.12	0.317	0.363	1.09	0.275	0.321
Estimated Intracranial Volume	**0.82**	**5.58e-18**	**1.67e-17**	**0.84**	**1.52e-19**	**5.05e-19**	0.83	0.409	0.437
Subcortical Gray Matter Volume	**0.66**	**3.83e-10**	**8.32e-10**	**0.87**	**3.47e-22**	**1.46e-21**	**4.05**	**5.12e-05**	**8.71e-05**
Cortical Volume	**0.67**	**3.24e-10**	**7.28e-10**	**0.86**	**5.50e-22**	**2.17e-21**	**4.34**	**1.43e-05**	**2.57e-05**
Cerebral White Matter Volume	**0.32**	**0.007**	**0.010**	**0.92**	**1.20e-28**	**7.74e-28**	**8.46**	**2.69e-17**	**7.69e-17**
Total Brain Volume	**0.60**	**4.57e-08**	**9.60e-08**	**0.96**	**3.64e-38**	**3.82e-37**	**8.38**	**5.52e-17**	**1.51e-16**

Top section: Left column shows Pearson correlations between standard single low-field (64mT) and high-field (3T) images for each brain measure. Middle column shows Pearson correlations between SynthSR-processed low-field and high-field estimates. Right column shows results of Steiger *Z*-tests, assessing whether the correlations for standard and SynthSR-processed scans are statistically different. Middle section: Left column shows Pearson correlations between standard single low-field (64mT) and high-field (3T) images for each brain measure. Middle column shows Pearson correlations between standard multi-orientation low-field and high-field estimates. Right column shows results of Steiger *Z*-tests, assessing whether the correlations for single and multi-orientation approaches are statistically different. Bottom section: Left column shows Pearson correlations between standard single low-field (64mT) and high-field (3T) images for each brain measure. Middle column shows Pearson correlations between SynthSR-processed multi-orientation low-field and high-field estimates. Right column shows results of Steiger *Z*-tests, assessing whether the correlations for standard and SynthSR-processed multi-orientation approaches are statistically different. Values in bold survive correction for multiple comparisons.

**Table 3:** Intraclass correlation coefficients (ICCs) for the correspondence between low-field (64mT) and high-field (3T) MR images and comparisons across super-resolution approaches.

Brain measure	Standard Axial 64mT ICCs with 3T			SynthSR-Processed Axial 64mT ICCs with 3T			Steiger’s		
	*ICC*	*p*	*q*	*ICC*	*p*	*q*	*Z*	*p*	*q*
Total Surface Area	**0.71**	**1.65e-12**	**4.15e-12**	**0.90**	**2.55e-26**	**1.46e-25**	**4.35**	**1.38e-05**	**2.41e-05**
Mean Cortical Thickness	−0.05	0.668	0.701	0.13	0.139	0.178	1.29	0.197	0.230
Estimated Intracranial Volume	**0.79**	**1.11e-16**	**3.04e-16**	**0.84**	**2.68e-20**	**1.06e-19**	**2.07**	**0.038**	**0.053**
Subcortical Gray Matter Volume	**0.66**	**1.46e-10**	**3.40e-10**	**0.88**	**1.14e-24**	**6.01e-24**	**4.57**	**4.81e-06**	**9.17e-06**
Cortical Volume	**0.61**	**1.08e-08**	**2.34e-08**	**0.82**	**1.32e-18**	**4.38e-18**	**4.06**	**4.91e-05**	**8.36e-05**
Cerebral White Matter Volume	**0.24**	**0.024**	**0.036**	**0.91**	**5.44e-29**	**3.43e-28**	**8.47**	**2.42e-17**	**7.34e-17**
Total Brain Volume	**0.56**	**1.43e-07**	**2.90e-07**	**0.97**	**1.49e-42**	**2.35e-41**	**9.40**	**5.49e-21**	**2.30e-20**
Brain measure	Standard Axial 64mT ICCs with 3T			Standard Multi-Orientation 64mT ICCs with 3T			Steiger’s		
	*ICC*	*p*	*q*	*ICC*	*p*	*q*	*Z*	*p*	*q*
Total Surface Area	**0.71**	**1.65e-12**	**4.15e-12**	**0.58**	**7.44e-08**	**1.56e-07**	**−1.88**	**0.059**	**0.080**
Mean Cortical Thickness	−0.05	0.668	0.701	−0.22	0.969	0.969	−1.32	0.187	0.223
Estimated Intracranial Volume	**0.79**	**1.11e-16**	**3.04e-16**	**0.76**	**7.51e-15**	**1.97e-14**	−1.94	0.052	0.072
Subcortical Gray Matter Volume	**0.66**	**1.46e-10**	**3.40e-10**	**0.71**	**2.32e-12**	**5.61e-12**	0.91	0.360	0.398
Cortical Volume	**0.61**	**1.08e-08**	**2.34e-08**	**0.49**	**7.32e-06**	**1.36e-05**	−1.53	0.126	0.165
Cerebral White Matter Volume	**0.24**	**0.024**	**0.036**	**0.30**	**0.006**	**0.010**	2.11	0.035	0.051
Total Brain Volume	**0.56**	**1.43e-07**	**2.90e-07**	**0.66**	**1.98e-10**	**4.46e-10**	**3.38**	**7.27e-04**	**0.001**
Brain measure	Standard Axial 64mT ICCs with 3T			SynthSR-Processed Multi-Orientation 64mT ICCs with 3T			Steiger’s		
	*ICC*	*p*	*q*	*ICC*	*p*	*q*	*Z*	*p*	*q*
Total Surface Area	**0.71**	**1.65e-12**	**4.15e-12**	**0.87**	**3.67e-23**	**1.78e-22**	**3.50**	**4.72e-04**	**7.62e-04**
Mean Cortical Thickness	−0.05	0.668	0.701	0.12	0.160	0.194	1.07	0.282	0.324
Estimated Intracranial Volume	**0.79**	**1.11e-16**	**3.04e-16**	**0.83**	**2.01e-19**	**7.04e-19**	1.45	0.147	0.185
Subcortical Gray Matter Volume	**0.66**	**1.46e-10**	**3.40e-10**	**0.86**	**2.58e-22**	**1.16e-21**	**3.91**	**9.17e-05**	**1.52e-04**
Cortical Volume	**0.61**	**1.08e-08**	**2.34e-08**	**0.84**	**5.64e-20**	**2.09e-19**	**4.45**	**8.54e-06**	**1.54e-05**
Cerebral White Matter Volume	**0.24**	**0.024**	**0.036**	**0.92**	**2.41e-29**	**1.69e-28**	**8.47**	**2.45e-17**	**7.34e-17**
Total Brain Volume	**0.56**	**1.43e-07**	**2.90e-07**	**0.96**	**1.56e-38**	**1.64e-37**	**8.43**	**3.42e-17**	**9.80e-17**

Top section: Left column shows ICC values between standard single low-field (64mT) and high-field (3T) images for each brain measure. Middle column shows ICCs between SynthSR-processed low-field and high-field estimates. Right column shows results of Steiger *Z*-tests, assessing whether the ICCs for standard and SynthSR-processed scans are statistically different. Middle section: Left column shows ICCs between standard single low-field (64mT) and high-field (3T) images for each brain measure. Middle column shows ICCs between standard multi-orientation low-field and high-field estimates. Right column shows results of Steiger *Z*-tests, assessing whether the ICCs for single and multi-orientation approaches are statistically different. Bottom section: Left column shows ICCs between standard single low-field (64mT) and high-field (3T) images for each brain measure. Middle column shows ICCs between SynthSR-processed multi-orientation low-field and high-field estimates. Right column shows results of Steiger *Z*-tests, assessing whether the ICCs for standard and SynthSR-processed multi-orientation approaches are statistically different. Values in bold survive correction for multiple comparisons.

At the regional level, we found moderate correspondence across widespread cortical regions for surface area (mean Pearson’s *r* across all 68 regions = 0.35, range = −0.23–0.62; mean ICC = 0.34, range = −0.16–0.59) and cortical volume (mean *r* across all 68 regions = 0.32, range = −0.24–0.64; mean ICC = 0.31, range − 0.21–0.58), whereas estimates for cortical thickness were either poor, null or negative (mean *r* across all 68 regions = −0.04, range = −0.37–0.33; mean ICC = −0.03, range = −0.31–0.29, Figure 2, Supplementary Tables 2, 3). In particular, the brainstem (*r* = 0.79, *p* = 3.29e-16, *q* = 7.21e-14, ICC = 0.78, *p* = 2.74e-16, *q* = 4.29e-15) and lateral ventricle (right, *r* = 0.92, *p* = 3.67e-29, *q* = 6.67e-27; ICC = 0.92, *p* = 1.53e-29, *q* = 2.79e-27; left, *r* = 0.96, *p* = 9.47e-38, *q* = 2.58e-35; ICC = 0.95, *p* = 2.89e-36, *q* = 7.86e-34) low-field estimates showed high fidelity to measures from high-field scans (Figures 2, 3A). For surface area and cortical volume, the strongest positive correlations were observed in prefrontal (area, left rostral middle frontal, *r* = 0.61, *p* = 2.61e-08, *q* = 1.31e-07; ICC = 0.56, *p* = 2.02e-07, *q* = 9.38e-07), occipital (volume, right lateral occipital, *r* = 0.57, *p* = 3.13e-07, *q* = 1.36e-06; ICC = 0.56, *p* = 1.46e-07, *q* = 6.91e-07) and right anterior cingulate regions (area, *r* = 0.62, *p* = 9.78e-09, *q* = 5.24e-08; ICC = 0.49, *p* = 8.19e-06, *q* = 2.97e-05; volume, *r* = 0.64, *p* = 2.45e-09, *q* = 1.39e-08; ICC = 0.58, *p* = 6.08e-08, *q* = 2.99e-07; see Supplementary Tables 2, 3).

### Application of SynthSR significantly improves correspondence between neuroimaging measures extracted from single orientation low-field scans and high-field scans

When we applied SynthSR to single T1/T2 pairs of low-field-acquired images, we found significantly improved correspondence with high-field images for most global brain measures, with the exception of mean cortical thickness and intracranial volume (see Tables 2, 3). We observed statistically significant improvements for global estimates of cerebral white matter volume (standard image Pearson’s *r* = 0.32, SynthSR-processed image *r* = 0.92; Steiger’s *z* = 8.75, *p* = 2.18e-18, *q* = 6.88e-18), total brain volume (standard *r* = 0.60, SynthSR-processed *r* = 0.97; *z* = 9.80, *p* = 1.15e-22, *q* = 5.59e-22), cortical volume (standard *r* = 0.67, SynthSR-processed *r* = 0.86; *z* = 4.18, *p* = 2.90e-05, *q* = 5.08e-05), subcortical gray matter volume (standard *r* = 0.66, SynthSR-processed *r* = 0.88; *z* = 4.57, *p* = 4.84e-06, *q* = 8.97e-06) and total surface area (standard *r* = 0.76, SynthSR-processed *r* = 0.90; *z* = 3.73, *p* = 1.94e-04, *q* = 3.22e-04). With the exception of cortical thickness, the ICC also showed significant improvements in correspondence, with ICC coefficients for SynthSR-processed images in the range of 0.84–0.97 (Table 3). When we examined the individual data distributions (Figure 4), we found that processing low-field scans with SynthSR improved correspondence by increasing global brain estimates to more closely approximate the high-field data. Mean differences (SynthSR subtracting 3T) for estimates of surface area were −0.4% (SD 4.1), cortical volume −19.4% (4.4), subcortical volume −11.4% (4.2), cerebral white matter volume + 1.8% (5.6), total brain volume −9.4% (2.3) and estimated intracranial volume + 1.3% (7.2). However, for cortical thickness, SynthSR decreased global estimates, resulting in poorer correspondence with high-field values (mean difference for standard low-field 0.85% [5.4], mean difference for SynthSR-processed low-field −12.9% [3.7]). Bland–Altman plots of agreement (see Supplementary Figure 3) showed positive bias for total brain volume (1.14e05 mm^3^, 95% limits of agreement [0.52e5-1.75e5]), cortical volume (1.02e05 mm^3^ [0.44e05-1.59e05]), subcortical volume (2.12e04 mm^3^ [1.94e3-12.98e3]), and cortical thickness (0.35 mm [0.14–0.56]), indicating that SynthSR-processed low-field images still underestimated 3T data. There was no or minimal bias for total surface area (977 mm^2^, 95% limits of agreement −1.65e04–1.85e04), intracranial volume (−1.29e04 mm^3^ [−2.09e05–1.83e05]), and cerebral white matter volume (−7.03e03 mm^3^ [−55.96e03–41.89e03]). At the regional level, processing with SynthSR significantly improved cortical thickness correspondence (via Pearson correlations) within temporal (*z* range = 2.18–4.08; Figure 3B and 3C; Supplementary Table 2) and prefrontal regions (*z* range = 2.78–2.85), with similar improvements when assessed via ICC (temporal z range 2.65–4.02; prefrontal z range 2.44–2.68; Supplementary Table 3). In addition, using SynthSR yielded widespread improvements in image correspondence (assessed via Pearson correlations) for regional estimates of cortical surface area (mean *z* = 2.55, range = −0.85–5.70), cortical volume (mean *z* = 2.51, range = −0.55–6.18) and subcortical volume (mean *z* = 2.50, range = −1.67–5.15), with the strongest effects observed in the left superior temporal lobe (area, *z* = 5.45; volume, *z* = 6.18) and precentral gyri supplementary motor areas (area, *z* = 5.70; volume, *z* = 5.42; Figure 3C; Supplementary Table 2; corresponding ICC values can be found in Supplementary Table 3).

### A multi-orientation approach achieves modest improvements on a single-orientation approach for some neuroimaging measures

When we used a multi-orientation (*N* = six pairs of T1/T2 multi-orientation scans) image averaging approach, we found modest and significantly improved correspondence to high-field scans for global estimates of cerebral white matter volume as assessed via Pearson’s correlation (*z* = 3.57, *p* = 3.64e-04, *q* = 5.73e-04) and total brain volume (*z* = 3.49, *p* = 4.84e-04, *q* = 7.44e-04; Table 2). In contrast, we found a negative relationship for mean cortical thickness, with higher cortical thickness values in the 3T corresponding to lower total surface area (*z* = −2.52, *p* = 0.012, *q* = 0.016) and estimated intracranial volume (*z* = −2.36, *p* = 0.018, *q* = 0.024; Table 2) values in the low-field data. We did not see a significant improvement in global estimates of cortical thickness, subcortical and cortical volume when we increased the number of scan acquisitions. Examination of the data distributions for cerebral white matter and total brain volume (Supplementary Figure 4) revealed that the multi-orientation approach increased the correspondence for all scans in a relatively uniform manner. In contrast, there were highly varied outcomes for the remaining brain measures (i.e., surface area, cortical thickness, subcortical and cortical gray matter volume), whereby some scans improved while others decreased in correspondence to the high-field estimates.

At the regional level, when we used a multi-orientation approach, correspondence (via Pearson correlations) became more negative for cortical thickness in several brain regions, including the left rostral middle frontal (*z* = −2.30, *p* = 0.021, *q* = 0.040) and bilateral supramarginal gyri (left, *z* = −2.69, *p* = 0.007, *q* = 0.015; right, *z* = −3.57, *p* = 3.63e-04, *q* = 9.60e-04; Figure 3D and 3E; Supplementary Table 4; corresponding ICC values can be found in Supplementary Table 5). Correlations also became significantly more negative in the right middle temporal gyrus for estimates of cortical surface area (*z* = −2.26, *p* = 0.024, *q* = 0.044) and cortical volume (*z* = −2.23, *p* = 0.026, *q* = 0.047).

### A combination of SynthSR and multi-orientation approaches achieved significant improvements in image correspondence over a standard single-pair acquisition

When we combined the multi-orientation (*N* = six T1/T2 multi-orientation scan pairs) and SynthSR processing approaches, we found significantly improved correspondence compared to a standard single-pair acquisition for almost all brain measures (Tables 2, 3). Following this combined approach, almost all global measures showed excellent correspondence (Pearson *r*’s > 0.85, ICC values >0.84) with high-field images, with the exception of mean cortical thickness, of which correspondence was not significantly improved following additional processing (Pearson’s *r* = 0.12, *p* = 0.317, *q* = 0.363; ICC = 0.12, *p* = 0.160, *q* = 0.194). We found that the combined approach resulted in uniform increases in global estimates across all individuals, with cortical thickness as the exception (Supplementary Figure 5). At a regional level, the combined approach improved correspondence for surface area and volume across widespread cortical and subcortical regions, with strongest effects observed in the temporal lobe and supplementary motor areas (Figure 3F and 3G).

### Multi-orientation scans processed with SynthSR improved image correspondence over single-acquisition scans processed with SynthSR in localized regions of the cortex

Finally, we compared the difference in correspondence between single-acquisition (*N* = one T1/T2 pair) scans processed with SynthSR and multi-orientation (*N* = six T1/T2 pairs) scans processed with SynthSR. We found that this approach decreased the correspondence for estimates of global surface area (*z* = −2.16, *p* = 0.031, *q* = 0.042), but did not significantly influence other global measures (Supplementary Table 6). At a regional level, increasing the number of scans acquired and processed with SynthSR improved the correspondence of regions primarily within the cingulate cortex (Supplementary Figure 6).

### Correlations with age and motion

We tested whether age or motion were associated with individual-level differences between low- and high-field estimates of each brain measure. After correction for multiple comparisons, there were no statistically significant relationships between the individual-level differences in high- and low-field data and participant motion (See Supplementary Table 7). Age was negatively correlated with discrepancies in estimates of cortical thickness following processing with SynthSR (Pearson’s *r* = −0.59, *p* = 8.36e-08, *q* = 2.73e-06; Supplementary Table 8), and was also negatively correlated with discrepancies in estimates of cortical volume across all processing approaches. This indicates that the younger the participant, the greater the discrepancy between low-field and the 3T estimates. Further, we observed a negative correlation between age and discrepancies in estimates of surface area in the standard acquisition (Pearson’s *r* = −0.59, *p* = 8.36e-08, *q* = 2.73e-06), which was not present in images processed with SynthSR.

## Discussion

In this study, we evaluated the correspondence between MR images acquired from low- and high-field scanners in a community sample of young people. We found that pairs of T1- and T2-weighted images obtained at low-field (64mT) showed high fidelity to images acquired at high-field (3T) for most global and regional brain measures, most notably measures of surface area, cortical volume, and subcortical volume. When we implemented a novel super-resolution method via a CNN [i.e., SynthSR, (44)], we found significantly improved image correspondence across almost all brain measures. When we increased the number of low-field scan acquisitions and orientations, we observed improvements in low-field image correspondence for estimates of cerebral white matter volume and total brain volume. The discrepancy between low-field and 3T measurements was not related to motion during the low-field scan session. These results demonstrate the potential of low-field imaging in young people and provide a foundation for future work to further improve low-field image quality and fidelity.

We found that standard single-acquisition low-field scans showed high correspondence with high-field-acquired scans for estimates of intracranial volume (*r* = 0.82) and surface area (*r* = 0.76), and moderate correspondence for estimates of subcortical, cortical gray and total brain volume (*r* = 0.60–0.67). These findings support previous work in children and adolescents demonstrating high fidelity between low-field and high-field scans for estimates of global gray matter, white matter, and total intracranial volume (22). Further, we extend previous work by characterizing the regional variation in LF-3T correspondence in cortical surface area, cortical volume, and subcortical volume, identifying regions with the greatest fidelity to high-field scans. Regions with the strongest correlations between low- and high-field tended to be larger and are typically high-contrast (i.e., the brainstem and ventricles), consistent with previous work demonstrating greater image fidelity for low-field data within larger brain regions (33). Within cortical regions, the greater correspondence within temporal, prefrontal, and cingulate lobes are important developmentally as these regions show protracted maturational trajectories (51, 52) and are commonly implicated in mental health disorders (53). The considerably lower cost and increased accessibility offered by low-field technology, coupled with demonstrated correspondence with high-field scans, provide a compelling argument for implementation of low-field technology at scale among young people.

We found that processing scans via SynthSR significantly improved image correspondence for global and regional estimates of gray and white matter volume, subcortical volume, surface area and total brain volume in young people. Regions that showed the greatest improvements included temporal and prefrontal regions, parts of the brain which have a developmental peak last, as opposed to sensory and motor cortices, which reach maturation at earlier points in development (54, 55). Our findings build on and extend recent work in adults demonstrating high correspondence between low- and high-field images following processing with SynthSR (44). While no incidental findings were reported in this study, future work should seek to investigate how SynthSR and other super-resolution methods might help to improve the ability to identify these findings in clinical and non-clinical samples.

We found that individual-level differences in estimates between low-field and high-field data were negatively correlated with age for cortical thickness and cortical volume several brain measures, indicating that with both standard and SynthSR-processed low-field estimates data were more accurate in older individuals for these measures. This might be partly explained by the existing reference library used to build SynthSR, which was built on an adult sample (44). Alternatively, age-associated patterns in gray-white matter contrast may contribute to the ability to differentiate the pial surface and white matter boundary at differing ages (56, 57). However, on the whole, despite being derived from adult training data, SynthSR still performed quite well in improving image correspondence in our sample, which included youth from middle childhood, adolescents, and young adults. These results demonstrate the validity of using SynthSR in estimating global and regional cortical surface area, cortical volume, and subcortical volume measures in younger populations.

We found that implementing a multi-orientation scan series yielded modest improvements in image correspondence for estimates of cerebral white matter and total brain volume. However, these improvements were not observed in other brain measures. There was decreased correspondence for estimates of surface area and intracranial volume, and null effects for cortical thickness. While our approach did not show the same degree of improvement displayed in previous work (31, 32), we note that these studies were acquired with a high-field (3T) magnet, in contrast to the low-field magnet used in our study.

It is also possible that we could employ alternative co-registration procedures to improve this correspondence, as we only implemented one type of co-registration in this study (i.e., an affine transformation registered to the axial image). In future, we may also achieve improved low-field and 3T measure correspondence if we employ alternative multi-orientation approaches. These approaches might include, for example, combining a multi-orientation approach with variable echo times (58), or obtaining multiple scans in a single orientation (59). The variable results we observed across brain measures may suggest that specific super-resolution approaches might be more effective for specific brain tissues — further research is necessary to investigate this possibility.

In this sample, neither super-resolution approach improved the correspondence of low-field estimates for total mean cortical thickness. However, at the regional level, processing with SynthSR yielded significant improvements in correspondence within the prefrontal and temporal lobes; for example, correspondence increased from *r* = −0.18 to *r* = 0.43 within the right temporal lobe following processing with SynthSR. As cortical thickness is measured at the sub-millimeter level, changes in voxel size can greatly affect those measurements. As a result, estimation of cortical thickness remains challenging at low field, whereby images are acquired with larger voxel size and slice thickness than traditional high-field-strength systems (44). In addition, the lower contrast-to-noise ratio of low-field images increases the difficulty for software to accurately estimate gray and white matter boundaries, which are required for accurate estimation of cortical thickness (45). In order to address these challenges, further refinement of acquisition and processing pipelines, or deployment of alternative super-resolution approaches, will be necessary to successfully recapitulate estimates of cortical thickness in low-field-strength systems. We are rigorously investigating additional methods at this time. Improvements to the measurement of cortical thickness with low-field MRI are important for implementation of low-field MR systems among youth and young adults, given the marked changes in cortical thickness that occur during this developmental period (25, 60) and its relevance to several psychiatric disorders (61, 62). One approach may be to leverage the advantages of both low- and high-strength MRI systems by employing a sequential staging approach, using low-field systems at scale for initial identification of high-risk cases, and reserving high-field MR for later classification of true-positive from false-positive findings (63). Alternatively, one promising approach may be to use measurement-in-error statistical modeling. In other fields of medicine, measurement-in-error statistical modeling is often used to develop low-cost, convenient measures for risk assessment (64–66). Firstly, a functional relationship is established between a ‘gold standard’ measure (e.g., 3T-acquired image) and a noisier, low-cost, convenient measure (e.g., LF-acquired image) for risk assessment (67). This model is then transported to an external sample where a proxy estimate of the gold standard measure is obtained using only the noisier measure. We plan to test the feasibility of this approach once we have collected sufficient data in an independent sample of youth.

Characterization of the diverse neurodevelopmental trajectories in mental health and illness is integral to understanding the etiology of mental disorders in youth. Despite the advent of ‘big data’ and the emergence of several large neuroimaging datasets, most samples only include individuals who can travel to fixed MR scanners within urban medical and research centers (68, 69), commonly termed “samples of convenience.” Systemic and structural barriers prohibiting widespread MR access limit the diversity and generalizability of neuroimaging samples, which may act to reinforce systemic biases in inference and interpretation (68). Low-field technology offers an opportunity to improve the accessibility of MR technology to underserved populations, thereby addressing systemic biases in healthcare access and in representation within neuroscience (68, 69). Also, for neuroimaging to have practical prognostic and diagnostic utility, we must be able to obtain MRI scans in a variety of settings, not only in urban research centers. Incorporating community settings into risk assessment programs is crucial to identify at-risk individuals earlier, reach individuals traditionally underserved by medical research centers, and progress toward the goal of universal screening and prevention (70–72). This approach will also satisfy service users’ preference for community settings and easily implemented assessments (72). Finally, in young people in particular, increasing diversity in neuroscience is essential to improving our ability to understand inter-individual differences in brain development, identify emerging neurodevelopmental and psychiatric disorders, and discover biomarkers that are generalizable to diverse populations (68, 73).

We must acknowledge the limitations of this study. This study was conducted in healthy children, adolescents, and young adults; further work is necessary to test the generalizability of these findings to clinical populations. Further, we assumed high-field acquired images as the “ground truth,” although the accuracy of high-field MR images in estimating structural brain measures has not been established. While we used pairs of T1- and T2-weighted images, previous low-field studies have used only T2-weighted scans (22); thus, additional work is necessary to quantitatively compare and evaluate these contrasting approaches. We did not apply distortion correction or concomitant field correction for either low-field or high-field scans; employing these correction strategies may help to further improve image quality, as has been demonstrated elsewhere (74). In addition, our selected acquisition parameters and image processing and super-resolution software were not specifically developed for pediatric populations; developmentally-specific software has yet to be developed, and may further improve image fidelity and quality.

In summary, we found that brain structural images acquired in a sample of young people in a portable low-field MR system showed high fidelity to high-field MR images for measures of brain volume and surface area. Additionally, we found that greater correspondence to high-field images could be achieved for cerebral white matter volume and subcortical volume following processing via a CNN developed for low-field images. In contrast, using a multi-orientation image averaging approach resulted in modest improvements in image correspondence for measures of white matter volume and total brain volume, but resulted in lower correspondence for surface area and intracranial volume. Finally, we found that using a combined multi-orientation and CNN-processing approach significantly improved image correspondence when compared to standard single-acquisition scans, but negligible improvements in correspondence above single-acquisition, CNN-processed scans. Taken together, our results indicate that using single pairs of T1- and T2-weighted images, combined with super-resolution of images via SynthSR, yielded the greatest improvements in correspondence between low-field and high-field MR images. Future work should seek to evaluate different combinations of acquisition and processing approaches to further improve images acquired with portable low-field systems.

## Data availability statement

The raw data supporting the conclusions of this article will be made available by the authors, without undue reservation.

## Ethics statement

The studies involving humans were approved by Institutional Review Board, Boston Children’s Hospital. The studies were conducted in accordance with the local legislation and institutional requirements. Written informed consent for participation in this study was provided by the participants’ legal guardians/next of kin.

## Author contributions

RC: Formal analysis, Methodology, Writing – original draft, Writing – review & editing. RH: Data curation, Formal analysis, Investigation, Methodology, Visualization, Writing – original draft, Writing – review & editing. MC: Data curation, Methodology, Writing – review & editing. KS: Writing – review & editing. TA: Methodology, Resources, Writing – review & editing. JS: Methodology, Resources, Writing – review & editing. DG: Conceptualization, Investigation, Methodology, Project administration, Resources, Supervision, Writing – review & editing. MJ: Conceptualization, Data curation, Formal analysis, Funding acquisition, Investigation, Methodology, Project administration, Resources, Supervision, Visualization, Writing – original draft, Writing – review & editing.
